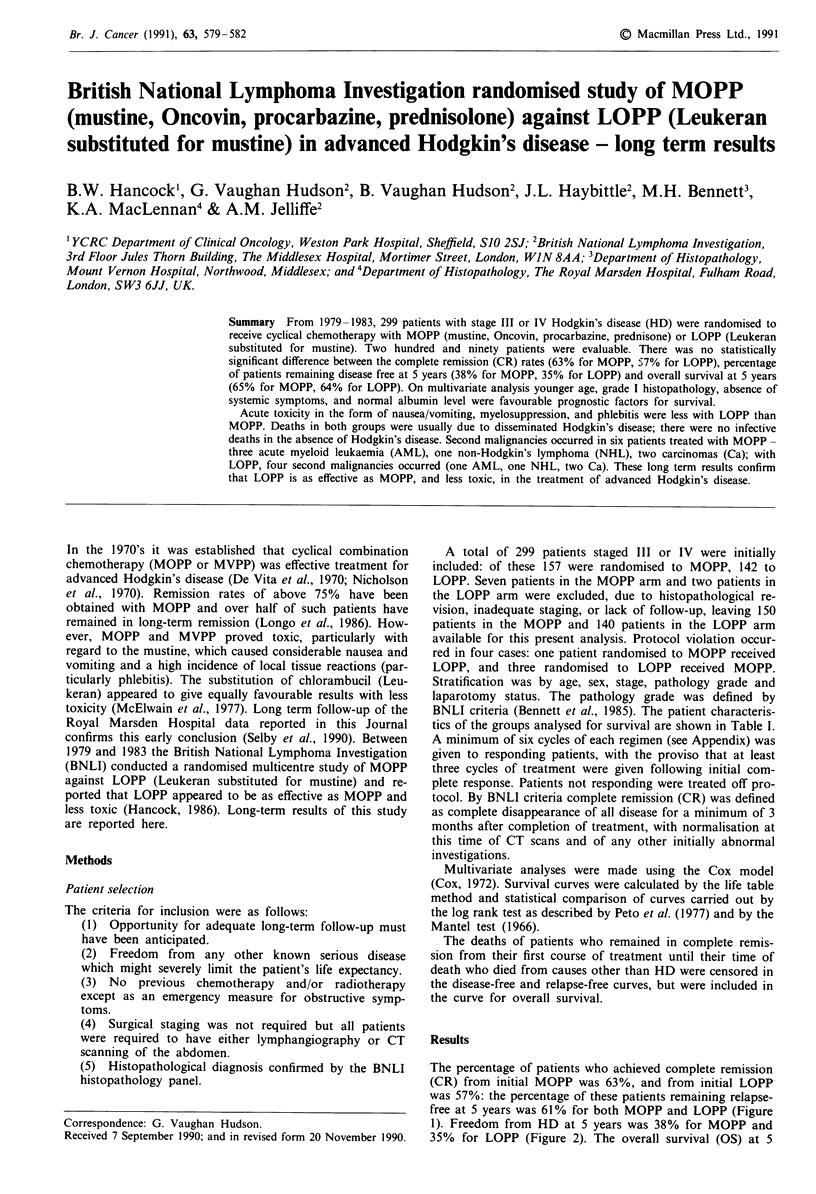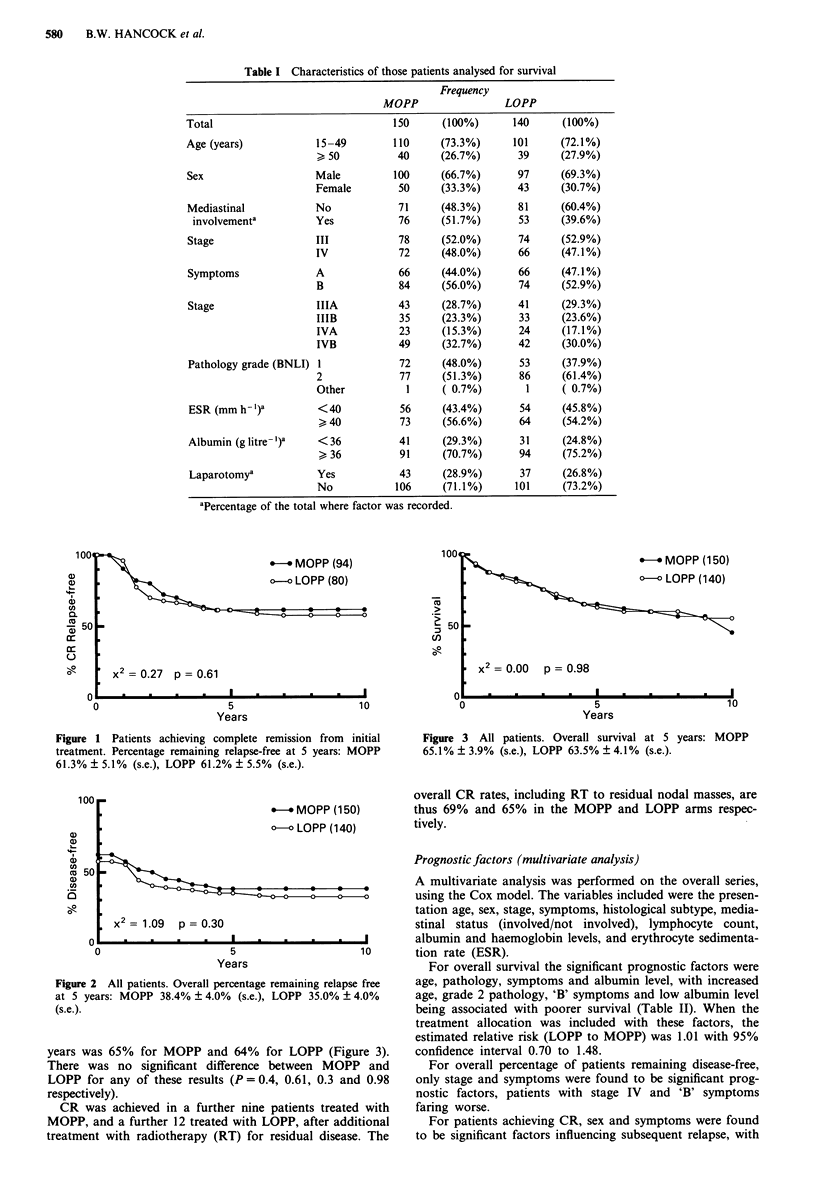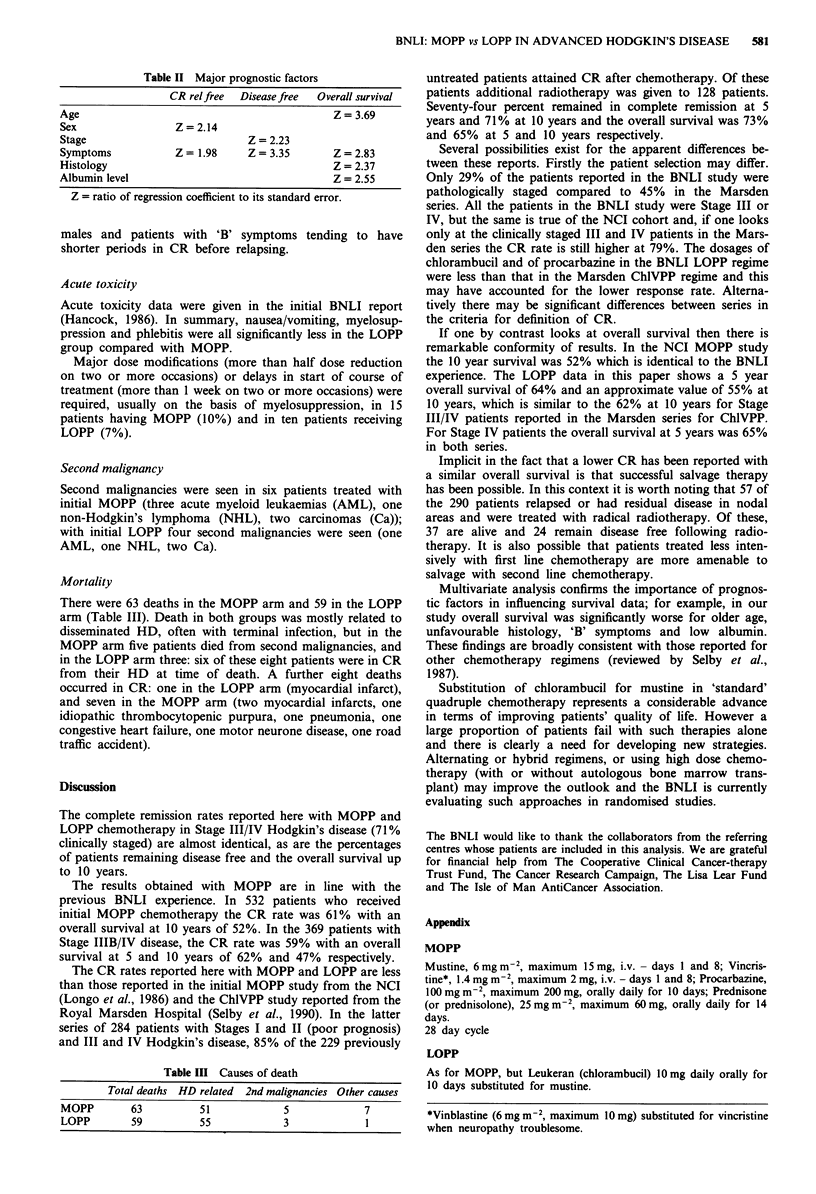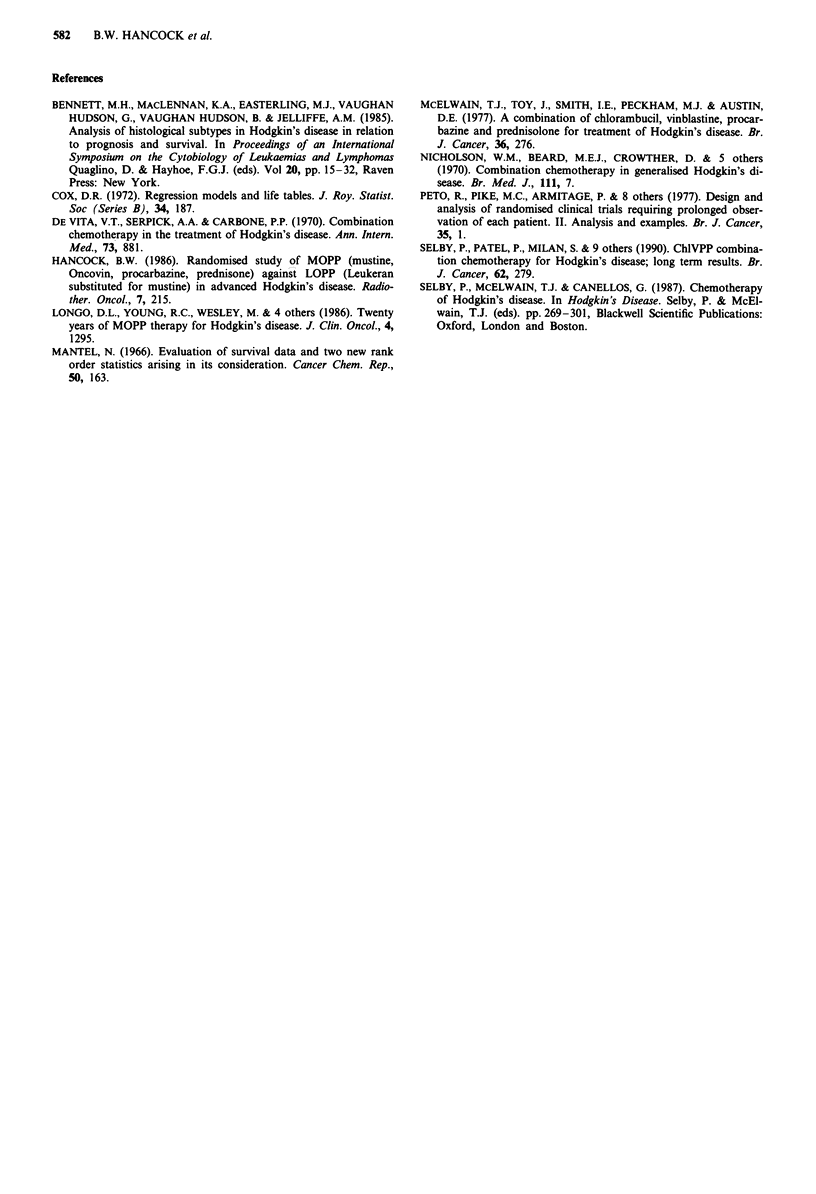# British National Lymphoma Investigation randomised study of MOPP (mustine, Oncovin, procarbazine, prednisolone) against LOPP (Leukeran substituted for mustine) in advanced Hodgkin's disease--long term results.

**DOI:** 10.1038/bjc.1991.134

**Published:** 1991-04

**Authors:** B. W. Hancock, G. Vaughan Hudson, B. Vaughan Hudson, J. L. Haybittle, M. H. Bennett, K. A. MacLennan, A. M. Jelliffe

**Affiliations:** YCRC Department of Clinical Oncology, Weston Park Hospital, Sheffield, UK.

## Abstract

From 1979-1983, 299 patients with stage III or IV Hodgkin's disease (HD) were randomised to receive cyclical chemotherapy with MOPP (mustine, Oncovin, procarbazine, prednisone) or LOPP (Leukeran substituted for mustine). Two hundred and ninety patients were evaluable. There was no statistically significant difference between the complete remission (CR) rates (63% for MOPP, 57% for LOPP), percentage of patients remaining disease free at 5 years (38% for MOPP, 35% for LOPP) and overall survival at 5 years (65% for MOPP, 64% for LOPP). On multivariate analysis younger age, grade I histopathology, absence of systemic symptoms, and normal albumin level were favourable prognostic factors for survival. Acute toxicity in the form of nausea/vomiting, myelosuppression, and phlebitis were less with LOPP than MOPP. Deaths in both groups were usually due to disseminated Hodgkin's disease; there were no infective deaths in the absence of Hodgkin's disease. Second malignancies occurred in six patients treated with MOPP--three acute myeloid leukaemia (AML), one non-Hodgkin's lymphoma (NHL), two carcinomas (Ca); with LOPP, four second malignancies occurred (one AML, one NHL, two Ca). These long term results confirm that LOPP is as effective as MOPP, and less toxic, in the treatment of advanced Hodgkin's disease.


Br. J. Cancer (1991), 63, 579-582 © Macmillan Press Ltd., 1991

### British National Lymphoma Investigation randomised study of MOPP (mustine, Oncovin, procarbazine, prednisolone) against LOPP (Leukeran substituted for mustine) in advanced Hodgkin's disease – long term results

B.W. Hancock[1], G. Vaughan Hudson[2], B. Vaughan Hudson[2], J.L. Haybittle[2], M.H. Bennett[3], K.A. MacLennan[4] & A.M. Jelliffe[2]

[1]*YCRC Department of Clinical Oncology, Weston Park Hospital, Sheffield, S10 2SJ;* [2]*British National Lymphoma Investigation, 3rd Floor Jules Thorn Building, The Middlesex Hospital, Mortimer Street, London, W1N 8AA;* [3]*Department of Histopathology, Mount Vernon Hospital, Northwood, Middlesex; and* [4]*Department of Histopathology, The Royal Marsden Hospital, Fulham Road, London, SW3 6JJ, UK.*

**Summary** From 1979–1983, 299 patients with stage III or IV Hodgkin's disease (HD) were randomised to receive cyclical chemotherapy with MOPP (mustine, Oncovin, procarbazine, prednisone) or LOPP (Leukeran substituted for mustine). Two hundred and ninety patients were evaluable. There was no statistically significant difference between the complete remission (CR) rates (63% for MOPP, 57% for LOPP), percentage of patients remaining disease free at 5 years (38% for MOPP, 35% for LOPP) and overall survival at 5 years (65% for MOPP, 64% for LOPP). On multivariate analysis younger age, grade I histopathology, absence of systemic symptoms, and normal albumin level were favourable prognostic factors for survival.

Acute toxicity in the form of nausea/vomiting, myelosuppression, and phlebitis were less with LOPP than MOPP. Deaths in both groups were usually due to disseminated Hodgkin's disease; there were no infective deaths in the absence of Hodgkin's disease. Second malignancies occurred in six patients treated with MOPP – three acute myeloid leukaemia (AML), one non-Hodgkin's lymphoma (NHL), two carcinomas (Ca); with LOPP, four second malignancies occurred (one AML, one NHL, two Ca). These long term results confirm that LOPP is as effective as MOPP, and less toxic, in the treatment of advanced Hodgkin's disease.

In the 1970's it was established that cyclical combination chemotherapy (MOPP or MVPP) was effective treatment for advanced Hodgkin's disease (De Vita *et al.*, 1970; Nicholson *et al.*, 1970). Remission rates of above 75% have been obtained with MOPP and over half of such patients have remained in long-term remission (Longo *et al.*, 1986). However, MOPP and MVPP proved toxic, particularly with regard to the mustine, which caused considerable nausea and vomiting and a high incidence of local tissue reactions (particularly phlebitis). The substitution of chlorambucil (Leukeran) appeared to give equally favourable results with less toxicity (McElwain *et al.*, 1977). Long term follow-up of the Royal Marsden Hospital data reported in this Journal confirms this early conclusion (Selby *et al.*, 1990). Between 1979 and 1983 the British National Lymphoma Investigation (BNLI) conducted a randomised multicentre study of MOPP against LOPP (Leukeran substituted for mustine) and reported that LOPP appeared to be as effective as MOPP and less toxic (Hancock, 1986). Long-term results of this study are reported here.

### Methods

### *Patient selection*

The criteria for inclusion were as follows:

(1) Opportunity for adequate long-term follow-up must have been anticipated.

(2) Freedom from any other known serious disease which might severely limit the patient's life expectancy.

(3) No previous chemotherapy and/or radiotherapy except as an emergency measure for obstructive symptoms.

(4) Surgical staging was not required but all patients were required to have either lymphangiography or CT scanning of the abdomen.

(5) Histopathological diagnosis confirmed by the BNLI histopathology panel.

Correspondence: G. Vaughan Hudson.
Received 7 September 1990; and in revised form 20 November 1990.

A total of 299 patients staged III or IV were initially included: of these 157 were randomised to MOPP, 142 to LOPP. Seven patients in the MOPP arm and two patients in the LOPP arm were excluded, due to histopathological revision, inadequate staging, or lack of follow-up, leaving 150 patients in the MOPP and 140 patients in the LOPP arm available for this present analysis. Protocol violation occurred in four cases: one patient randomised to MOPP received LOPP, and three randomised to LOPP received MOPP. Stratification was by age, sex, stage, pathology grade and laparotomy status. The pathology grade was defined by BNLI criteria (Bennett *et al.*, 1985). The patient characteristics of the groups analysed for survival are shown in Table I. A minimum of six cycles of each regimen (see Appendix) was given to responding patients, with the proviso that at least three cycles of treatment were given following initial complete response. Patients not responding were treated off protocol. By BNLI criteria complete remission (CR) was defined as complete disappearance of all disease for a minimum of 3 months after completion of treatment, with normalisation at this time of CT scans and of any other initially abnormal investigations.

Multivariate analyses were made using the Cox model (Cox, 1972). Survival curves were calculated by the life table method and statistical comparison of curves carried out by the log rank test as described by Peto *et al.* (1977) and by the Mantel test (1966).

The deaths of patients who remained in complete remission from their first course of treatment until their time of death who died from causes other than HD were censored in the disease-free and relapse-free curves, but were included in the curve for overall survival.

### Results

The percentage of patients who achieved complete remission (CR) from initial MOPP was 63%, and from initial LOPP was 57%: the percentage of these patients remaining relapse-free at 5 years was 61% for both MOPP and LOPP (Figure 1). Freedom from HD at 5 years was 38% for MOPP and 35% for LOPP (Figure 2). The overall survival (OS) at 5

**Table I** Characteristics of those patients analysed for survival

| | | *Frequency* | | | |
|---|---|---|---|---|---|
| | | *MOPP* | | *LOPP* | |
| Total | | 150 | (100%) | 140 | (100%) |
| Age (years) | 15–49 | 110 | (73.3%) | 101 | (72.1%) |
| | ≥ 50 | 40 | (26.7%) | 39 | (27.9%) |
| Sex | Male | 100 | (66.7%) | 97 | (69.3%) |
| | Female | 50 | (33.3%) | 43 | (30.7%) |
| Mediastinal involvement[a] | No | 71 | (48.3%) | 81 | (60.4%) |
| | Yes | 76 | (51.7%) | 53 | (39.6%) |
| Stage | III | 78 | (52.0%) | 74 | (52.9%) |
| | IV | 72 | (48.0%) | 66 | (47.1%) |
| Symptoms | A | 66 | (44.0%) | 66 | (47.1%) |
| | B | 84 | (56.0%) | 74 | (52.9%) |
| Stage | IIIA | 43 | (28.7%) | 41 | (29.3%) |
| | IIIB | 35 | (23.3%) | 33 | (23.6%) |
| | IVA | 23 | (15.3%) | 24 | (17.1%) |
| | IVB | 49 | (32.7%) | 42 | (30.0%) |
| Pathology grade (BNLI) | 1 | 72 | (48.0%) | 53 | (37.9%) |
| | 2 | 77 | (51.3%) | 86 | (61.4%) |
| | Other | 1 | ( 0.7%) | 1 | ( 0.7%) |
| ESR (mm $h^{-1}$)[a] | <40 | 56 | (43.4%) | 54 | (45.8%) |
| | ≥ 40 | 73 | (56.6%) | 64 | (54.2%) |
| Albumin (g $litre^{-1}$)[a] | <36 | 41 | (29.3%) | 31 | (24.8%) |
| | ≥ 36 | 91 | (70.7%) | 94 | (75.2%) |
| Laparotomy[a] | Yes | 43 | (28.9%) | 37 | (26.8%) |
| | No | 106 | (71.1%) | 101 | (73.2%) |

[a]Percentage of the total where factor was recorded.

**Figure 1** Patients achieving complete remission from initial treatment. Percentage remaining relapse-free at 5 years: MOPP 61.3% ± 5.1% (s.e.), LOPP 61.2% ± 5.5% (s.e.).

**Figure 3** All patients. Overall survival at 5 years: MOPP 65.1% ± 3.9% (s.e.), LOPP 63.5% ± 4.1% (s.e.).

**Figure 2** All patients. Overall percentage remaining relapse free at 5 years: MOPP 38.4% ± 4.0% (s.e.), LOPP 35.0% ± 4.0% (s.e.).

years was 65% for MOPP and 64% for LOPP (Figure 3). There was no significant difference between MOPP and LOPP for any of these results ($P = 0.4$, 0.61, 0.3 and 0.98 respectively).

CR was achieved in a further nine patients treated with MOPP, and a further 12 treated with LOPP, after additional treatment with radiotherapy (RT) for residual disease. The overall CR rates, including RT to residual nodal masses, are thus 69% and 65% in the MOPP and LOPP arms respectively.

*Prognostic factors (multivariate analysis)*

A multivariate analysis was performed on the overall series, using the Cox model. The variables included were the presentation age, sex, stage, symptoms, histological subtype, mediastinal status (involved/not involved), lymphocyte count, albumin and haemoglobin levels, and erythrocyte sedimentation rate (ESR).

For overall survival the significant prognostic factors were age, pathology, symptoms and albumin level, with increased age, grade 2 pathology, 'B' symptoms and low albumin level being associated with poorer survival (Table II). When the treatment allocation was included with these factors, the estimated relative risk (LOPP to MOPP) was 1.01 with 95% confidence interval 0.70 to 1.48.

For overall percentage of patients remaining disease-free, only stage and symptoms were found to be significant prognostic factors, patients with stage IV and 'B' symptoms faring worse.

For patients achieving CR, sex and symptoms were found to be significant factors influencing subsequent relapse, with

**Table II** Major prognostic factors

| | *CR rel free* | *Disease free* | *Overall survival* |
|---|---|---|---|
| Age | | | Z = 3.69 |
| Sex | Z = 2.14 | | |
| Stage | | Z = 2.23 | |
| Symptoms | Z = 1.98 | Z = 3.35 | Z = 2.83 |
| Histology | | | Z = 2.37 |
| Albumin level | | | Z = 2.55 |

Z = ratio of regression coefficient to its standard error.

males and patients with 'B' symptoms tending to have shorter periods in CR before relapsing.

### *Acute toxicity*

Acute toxicity data were given in the initial BNLI report (Hancock, 1986). In summary, nausea/vomiting, myelosuppression and phlebitis were all significantly less in the LOPP group compared with MOPP.

Major dose modifications (more than half dose reduction on two or more occasions) or delays in start of course of treatment (more than 1 week on two or more occasions) were required, usually on the basis of myelosuppression, in 15 patients having MOPP (10%) and in ten patients receiving LOPP (7%).

### *Second malignancy*

Second malignancies were seen in six patients treated with initial MOPP (three acute myeloid leukaemias (AML), one non-Hodgkin's lymphoma (NHL), two carcinomas (Ca)); with initial LOPP four second malignancies were seen (one AML, one NHL, two Ca).

### *Mortality*

There were 63 deaths in the MOPP arm and 59 in the LOPP arm (Table III). Death in both groups was mostly related to disseminated HD, often with terminal infection, but in the MOPP arm five patients died from second malignancies, and in the LOPP arm three: six of these eight patients were in CR from their HD at time of death. A further eight deaths occurred in CR: one in the LOPP arm (myocardial infarct), and seven in the MOPP arm (two myocardial infarcts, one idiopathic thrombocytopenic purpura, one pneumonia, one congestive heart failure, one motor neurone disease, one road traffic accident).

### Discussion

The complete remission rates reported here with MOPP and LOPP chemotherapy in Stage III/IV Hodgkin's disease (71% clinically staged) are almost identical, as are the percentages of patients remaining disease free and the overall survival up to 10 years.

The results obtained with MOPP are in line with the previous BNLI experience. In 532 patients who received initial MOPP chemotherapy the CR rate was 61% with an overall survival at 10 years of 52%. In the 369 patients with Stage IIIB/IV disease, the CR rate was 59% with an overall survival at 5 and 10 years of 62% and 47% respectively.

The CR rates reported here with MOPP and LOPP are less than those reported in the initial MOPP study from the NCI (Longo *et al.*, 1986) and the ChlVPP study reported from the Royal Marsden Hospital (Selby *et al.*, 1990). In the latter series of 284 patients with Stages I and II (poor prognosis) and III and IV Hodgkin's disease, 85% of the 229 previously untreated patients attained CR after chemotherapy. Of these patients additional radiotherapy was given to 128 patients. Seventy-four percent remained in complete remission at 5 years and 71% at 10 years and the overall survival was 73% and 65% at 5 and 10 years respectively.

**Table III** Causes of death

| | *Total deaths* | *HD related* | *2nd malignancies* | *Other causes* |
|---|---|---|---|---|
| MOPP | 63 | 51 | 5 | 7 |
| LOPP | 59 | 55 | 3 | 1 |

Several possibilities exist for the apparent differences between these reports. Firstly the patient selection may differ. Only 29% of the patients reported in the BNLI study were pathologically staged compared to 45% in the Marsden series. All the patients in the BNLI study were Stage III or IV, but the same is true of the NCI cohort and, if one looks only at the clinically staged III and IV patients in the Marsden series the CR rate is still higher at 79%. The dosages of chlorambucil and of procarbazine in the BNLI LOPP regime were less than that in the Marsden ChlVPP regime and this may have accounted for the lower response rate. Alternatively there may be significant differences between series in the criteria for definition of CR.

If one by contrast looks at overall survival then there is remarkable conformity of results. In the NCI MOPP study the 10 year survival was 52% which is identical to the BNLI experience. The LOPP data in this paper shows a 5 year overall survival of 64% and an approximate value of 55% at 10 years, which is similar to the 62% at 10 years for Stage III/IV patients reported in the Marsden series for ChlVPP. For Stage IV patients the overall survival at 5 years was 65% in both series.

Implicit in the fact that a lower CR has been reported with a similar overall survival is that successful salvage therapy has been possible. In this context it is worth noting that 57 of the 290 patients relapsed or had residual disease in nodal areas and were treated with radical radiotherapy. Of these, 37 are alive and 24 remain disease free following radiotherapy. It is also possible that patients treated less intensively with first line chemotherapy are more amenable to salvage with second line chemotherapy.

Multivariate analysis confirms the importance of prognostic factors in influencing survival data; for example, in our study overall survival was significantly worse for older age, unfavourable histology, 'B' symptoms and low albumin. These findings are broadly consistent with those reported for other chemotherapy regimens (reviewed by Selby *et al.*, 1987).

Substitution of chlorambucil for mustine in 'standard' quadruple chemotherapy represents a considerable advance in terms of improving patients' quality of life. However a large proportion of patients fail with such therapies alone and there is clearly a need for developing new strategies. Alternating or hybrid regimens, or using high dose chemotherapy (with or without autologous bone marrow transplant) may improve the outlook and the BNLI is currently evaluating such approaches in randomised studies.

The BNLI would like to thank the collaborators from the referring centres whose patients are included in this analysis. We are grateful for financial help from The Cooperative Clinical Cancer-therapy Trust Fund, The Cancer Research Campaign, The Lisa Lear Fund and The Isle of Man AntiCancer Association.

### Appendix

### MOPP

Mustine, 6 mg $m^{-2}$, maximum 15 mg, i.v. – days 1 and 8; Vincristine*, 1.4 mg $m^{-2}$, maximum 2 mg, i.v. – days 1 and 8; Procarbazine, 100 mg $m^{-2}$, maximum 200 mg, orally daily for 10 days; Prednisone (or prednisolone), 25 mg $m^{-2}$, maximum 60 mg, orally daily for 14 days.
28 day cycle

### LOPP

As for MOPP, but Leukeran (chlorambucil) 10 mg daily orally for 10 days substituted for mustine.

*Vinblastine (6 mg $m^{-2}$, maximum 10 mg) substituted for vincristine when neuropathy troublesome.

### References

BENNETT, M.H., MACLENNAN, K.A., EASTERLING, M.J., VAUGHAN HUDSON, G., VAUGHAN HUDSON, B. & JELLIFFE, A.M. (1985). Analysis of histological subtypes in Hodgkin's disease in relation to prognosis and survival. In *Proceedings of an International Symposium on the Cytobiology of Leukaemias and Lymphomas* Quaglino, D. & Hayhoe, F.G.J. (eds). Vol **20**, pp. 15-32, Raven Press: New York.

COX, D.R. (1972). Regression models and life tables. *J. Roy. Statist. Soc (Series B)*, **34**, 187.

DE VITA, V.T., SERPICK, A.A. & CARBONE, P.P. (1970). Combination chemotherapy in the treatment of Hodgkin's disease. *Ann. Intern. Med.*, **73**, 881.

HANCOCK, B.W. (1986). Randomised study of MOPP (mustine, Oncovin, procarbazine, prednisone) against LOPP (Leukeran substituted for mustine) in advanced Hodgkin's disease. *Radiother. Oncol.*, **7**, 215.

LONGO, D.L., YOUNG, R.C., WESLEY, M. & 4 others (1986). Twenty years of MOPP therapy for Hodgkin's disease. *J. Clin. Oncol.*, **4**, 1295.

MANTEL, N. (1966). Evaluation of survival data and two new rank order statistics arising in its consideration. *Cancer Chem. Rep.*, **50**, 163.

MCELWAIN, T.J., TOY, J., SMITH, I.E., PECKHAM, M.J. & AUSTIN, D.E. (1977). A combination of chlorambucil, vinblastine, procarbazine and prednisolone for treatment of Hodgkin's disease. *Br. J. Cancer*, **36**, 276.

NICHOLSON, W.M., BEARD, M.E.J., CROWTHER, D. & 5 others (1970). Combination chemotherapy in generalised Hodgkin's disease. *Br. Med. J.*, **111**, 7.

PETO, R., PIKE, M.C., ARMITAGE, P. & 8 others (1977). Design and analysis of randomised clinical trials requiring prolonged observation of each patient. II. Analysis and examples. *Br. J. Cancer*, **35**, 1.

SELBY, P., PATEL, P., MILAN, S. & 9 others (1990). ChlVPP combination chemotherapy for Hodgkin's disease; long term results. *Br. J. Cancer*, **62**, 279.

SELBY, P., MCELWAIN, T.J. & CANELLOS, G. (1987). Chemotherapy of Hodgkin's disease. In *Hodgkin's Disease*. Selby, P. & McElwain, T.J. (eds). pp. 269-301, Blackwell Scientific Publications: Oxford, London and Boston.